# Case of Laceration of the Urethra without External Wound, Occasioned by Falling across the Edge of a Boat

**Published:** 1827-01

**Authors:** 

**Affiliations:** St. Thomas's Hospital.


*Case of Laceration of the Urethra without external Wound, occasioned by falling across the edge of a Boat.* Treated by Mr. GREEN, at ST. THOMAS'S HOSPITAL.

August 10th, 1826.—Francis Sawyer, ætatis forty-one, a stout muscular man, of healthy aspect, was admitted with a retention of urine of forty-eight hours' duration, the consequence of an injury to the perineum.

It appeared that, on the evening of August 8th, he was occupied in the chains of a vessel lying off Rotherhithe, when, by some accident, he lost his hold, and fell about six or seven feet, with one leg on each side of the edge of a boat, so that the perineum was severely bruised: considerable swelling and tension were the immediate consequences; but, having passed his urine two or three hours before, he felt no desire to evacuate the bladder.

During the night he slept well, as usual, and was quite easy till the morning of the 9th, when, on attempting to make water, he found that merely a small quantity of blood passed from the urethra, unmixed with urine. The retention continuing, and the desire to void the urine becoming more urgent, in the evening he applied to a surgeon, who attempted to introduce a catheter, but was foiled by the arrest of the instrument at the bulb of the urethra. About half a pint of blood flowed through the catheter. The surgeon then directed the application of twelve leeches and a poultice to the perineum. On August 10th, the urgency was still greater, and the lower part of the abdomen became painful; but his health was not disordered, and he was able to walk three or four hundred yards to consult another surgeon, who applied six leeches. On the evening of the 10th, he was brought to the hospital, when the perineum was found entire, but much swollen and tender to the touch; the penis and scrotum, and the inside of both thighs, discoloured by effused blood; and the bladder evidently much distended, producing considerable pain in the lower part of the abdomen. The desire to empty the bladder was very great, and gave an anxious expression to the countenance; the pulse was but slightly quickened.

Mr. Green, after attempting to introduce a catheter without success, (producing only a flow of blood,) directed the patient to be placed in the same posture as in the operation of lithotomy, and then made an incision in the line of the raphé of the perineum, the knife passing into a cell of blood, partly fluid, partly coagulated, extending towards the arch of the pubes. On introducing the catheter from the glans penis, it passed into this cell, protruding with it the ragged edges of the urethra, which was lacerated to the extent of an inch, probably close to the prostate gland, as no difficulty was experienced in passing the catheter on to the bladder. It was also distinctly seen that the triangular ligament was partially lacerated. Three pints of urine, highly tinged with blood, were drawn off, and the silver catheter left in the bladder, and fixed in its situation by a T bandage.

August 11th.—Has passed a comfortable night, and is easy and quite free from pain, excepting the smarting of the wound. Pulse sixty-five, full, soft, and regular; the tongue is foul, and he has slight thirst; the bowels have been moved twice; the urine passes through the catheter in tolerable quantity,—it is, however, mixed with blood: a small portion passes from the wound in the perineum. The tenderness of the abdomen has subsided.

The urine continued to be bloody till the 14th, after which it became natural. The bowels were rather costive, and he was ordered to take Ol. Ricini ℥ss. pro re nata. Some portion of the urine continued to pass by the wound till the 19th, after which the whole passed through the catheter.

August 20th.—The catheter having become partially plugged, it was removed, cleaned, and again introduced. The wound is healing rapidly, the granulations at the bottom having inosculated.

September 4th.—The granulations have filled the wound, and are now on the same level as the perineum. Cicatrisation to some extent has taken place at the extremity of the incision, and the same process is going on at the edges.

As the vessel to which he belonged was on the eve of sailing for Riga, and as the captain was unwilling to go without him, the patient was allowed to leave the hospital; having first exchanged the silver for an elastic gum catheter, which he was directed to wear till the wound was quite healed.

*Remarks.*—These cases, if not frequent, are not very rare. The treatment is obvious, and such as was employed in the histories above related. When the solution of continuity is complete, and the dissevered extremities are drawn half an inch asunder, some difficulty will be encountered in conveying a tube, whether of metal or elastic gum, into the bladder. In such event, the introduction of the latter instrument with a firm stilet into the vesical portion, and the gradual withdrawment of the stilet, so as to allow of a gentle increase of the curve of the catheter, greatly facilitates its progress into the bladder; a manœuvre which is practised in some other cases with advantage.

A circumstance connected with this accident is worthy of observation. Prominent swelling of the perineum and diffused discoloration are accompanied by an urgent desire, and an actual incapacity, to void the urine. A catheter cautiously introduced, when it reaches the arch of the pubes, gives the surgeon the impression of its being out of the canal, for its point is wholly unsupported, and falls from side to side. On withdrawing the stilet or the catheter, a free hemorrhage follows. The case sufficiently explains itself. But would not the surgeon, who was for the first time called to such a case, naturally enough suppose that the patient did not void his urine at the natural orifice, only because it escaped at the breach? If such were his apprehension, it would presently be removed, since the bladder becomes visibly and painfully distended, and all the symptoms proper to retention are manifested: and such is the case, whether the laceration is partial or amounts to a complete division of the tube. This invariable phenomenon offers a most fortunate escape from the dangerous consequences of a breach of the urethra, which is the result of distention from forcible efforts to pass urine in the case of stricture.

How is the retention to be explained? The interstice of the broken tube is plugged with coagula, and a quantity of fluid blood occupies the surrounding space, and forms a tense tumor in perineo; but this could be no impediment to the escape of the urine from a division or rent of the urethra, if the urine quitted the bladder. After laying open the perineum, and exposing the broken ends of the canal, the patient is quite incapable, by any effort, of expelling a drop of urine. If the bladder be wounded, nothing can prevent the escape of urine; and it is only in such ruptures of the urethra from external violence as are above detailed, that this symptom follows. A spasmodic contraction of the sphincter of the bladder, sympathetic with the injury, is the only satisfactory explanation of it. Retention of urine is among the many morbid sympathies with severe local injury, and in these casualties it is probable, that the default of consent occasioned by the laceration of the muscles ordinarily employed in the function of propelling the urine, is the proximate cause of the spasm. Thus the shock of the injury has the effect of averting a consequence which would tend greatly to aggravate the mischief.

Let us now briefly inquire what are the circumstances in which extravasation happens, and what those are which appear to favour or prevent this formidable consequence. It is almost needless to premise that we use this term not in its strict and literal sense, but as commonly used to imply diffusion through the cellular texture. Urine extravasated spoils and gangrenes all the cellular texture which it pervades. Distention, the stimulant quality of the fluid in its natural state, and its rapid decomposition, all contribute to the change which the cellular texture undergoes when loaded with it, and which is expressed by the French term "pourriture." Deep and extensive incisions, to relieve the integuments and facilitate the casting-off of the sloughs, constitute the principal local treatment. Acute abscess opening by ulceration into the urethra, and rent of the urethral membrane by distention behind a stricture, are the circumstances most frequently productive of extravasation. A breach or false passage, as it is termed, by the unskilful or imprudent use of an instrument, is an occasional but less frequent cause of the same accident. In this latter case, the obliquity of the false passage, and its opposite direction to that of the stream of urine,—the temporary hemorrhage, and obliteration of the breach by a coagulum,—the tendency of elastic structures, forcibly reft asunder, to close and contract when the separating force is withdrawn, and the adhesive process directly supervening, are circumstances which commonly protect the patient from the consequence of extravasation. But, in the case of rent or loss of substance from the process of ulceration, the ordinary cause of extravasation, the escape of urine is facilitated by its position on the bladder side of the stricture, as well as other circumstances of the breach. Much probably depends on the situation of the breach, in whatever way occasioned, not only as regards the stricture, but as regards the canal. A rupture of the membranous portion,—i. e. between the apex of the prostate gland and the bulb, (the commonest seat of stricture,) is more favourable to extravasation than a rupture of the prostatic or spongy portion of the urethra; and a rupture of that portion of the latter to which the septum scroti is attached, than of that which is anterior to the scrotum. The follicles and lacunæ of the prostatic urethra are subject to become dilated in old and firm strictures into pouches, having a valvular obliquity, under the pressure of an habitually loaded bladder and frequent forcible strainings: these rend, and suffer the urine to insinuate itself by little and little, and separate the membrane from its bed; and thus the diverticulum, becoming larger than the natural passage, yields to a protracted effort, and determines with apparent suddenness the result of extravasation. Now, from a pretty large experience of these cases we should say, that they are much more frequent among intemperate persons, who, having obstinate strictures, are neglectful of them, who occupy several minutes in passing a wire stream or dribbling drops of urine with extraordinary effort, and whose bladders become thickened and diminished in capacity,—than in persons who attempt to control the disease, and resolutely subject themselves to the use of instruments for comfort's sake, with all the hazard attached to their use indiscriminately.

The case of dilatation and rent or crevice behind the stricture, is unquestionably a more common origin of extravasation than that of abscess, acute or chronic, or false passage by instruments. Attentive observers must have remarked that persons who have fistulæ in perineo, or the vestiges of them, are comparatively seldom the subjects of extravasation of urine. The reason for this is self-evident. Fistula in perineo is nature's contrivance (sometimes, perhaps, a little aided by art,) for relieving the bladder suffering from a long existing permanent stricture. It is the ultimate state of a circumscribed abscess from chronic inflammation and condensation of the cellular tissue surrounding the strictured part. It is a false passage formed gradually, and therefore safely, upon the well-known principle in the animal economy, of thickening without, consentaneous to thinning within. The tendency of stricture to induce chronic inflammation and abscess in the surrounding texture, is universal throughout the canals of the body. The inflammation, condensation, induration, even to callosity, of the cellular membrane, and at length its suppuration, are distinct stages. Thus it happens in stricture of the intestine, both within and without the peritoneum,—of the œsophagus,—of the pylorus,—of the lacrymal and salivary ducts and ureters. Where the canal lies near the external surface, the abscess opens upon it, and gives relief, or avoids a fatal issue, by forming a fistula, or conduit for the delivery of the contents, whether secretory or excretory. When this is not the case, extravasation takes place into cavities or into the cellular substance, and in some of these instances proves fatal.

To conclude, then. Extravasation follows abscess opening into the urethra, or rent of the attenuated lining membrane in the expelling efforts, or extensive laceration of the canal by a wrong direction and violent use of instruments, when the provisional processes against extravasation are anticipated in point of time, or, if they exist, are insufficient. Chronic inflammation and circumscribed abscess are such processes, by the fistula thence resulting; and it may be added, that it is not occasional or absolute, so much as habitual and partial, but increasing retention, which leads to either termination.

Such are the pathological circumstances influencing the result of extravasation of urine. The mechanical are, shortly, the freedom or otherwise of the external opening, the direct or oblique course, and the backward or forward obliquity of the wound. In lithotomy, and bold incisions in perineo for the relief of the bladder, we incur no such risk; but a short wound of the urethra through the raphé scroti is followed by extravasation.

Another question of some interest, connected with these cases, is, whether the sexual act is impaired after a complete transverse division of the corpus spongiosum and urethra? and, if so, in what manner and degree? And this question is interesting in reference to operations upon the prostatic urethra and neck of the bladder, and especially that of lithotomy. This we must defer until another opportunity.

